# The Effects of Gender and Marital Status on Accrued Debt in Retirement Planning

**DOI:** 10.1093/geroni/igab046.930

**Published:** 2021-12-17

**Authors:** Karen Zurlo, Zibei Chen

**Affiliations:** 1 Rutgers University, Rutgers University, New Jersey, United States; 2 University of Southern Mississippi, Hattiesburg, Mississippi, United States

## Abstract

The effects of gender and marital status on accrued debt in retirement planning becomes an urgent concern because unmarried women face greater financial challenges in retirement than their counterparts. This study used data from the National Financial Capability Study (NFCS), designed by FINRA. We identified debt that influences retirement planning among a sample of pre-retirees, aged 51 to 61 years, and consider the associations of gender, marital status, debt, and retirement planning. Our results indicated that mortgage debt and credit card debt were negatively associated with retirement planning for women. Having a retirement account is positively associated with retirement planning and it also mediates the relationship between credit card debt and retirement planning. We urge women and financial planning executives to take time during the pre-retirement years to assess their various forms of debt and determine how it affects retirement planning objectives given current marital status.

